# Medicine as It Is

**Published:** 1855-09

**Authors:** Joseph P. Logan

**Affiliations:** Professor of Physiology and General Pathology in the Atlanta Medical College


ATLANTA

Medical and Surgical Journal.

VOL. I.] SEPTEMBER, 1855. [NO. 1.

### ORIGINAL COMMUNICATIONS.

ARTICLE 1.—*Medicine as it Is.* An essay presented to the Atlanta Medical Society, by appointment, and requested for publication by that body. By JOSEPH P. LOGAN, M. D.

Gentlemen of the Atlanta Medical Society:

I present myself before you, in obedience to the call which has been made upon me, though I feel that I have nothing to offer, which could be considered strictly, as a contribution to science—but as coming within the scope of what I suppose to be the objects of this Society, I shall bring to your attention, in a desultory way, some matters which I conceive to be intimately connected with the honor and interests of the Medical Profession, and specially appropriate to the circumstances in which we are placed.

I propose, briefly, to present some points connected with the claims of the art of healing, to be considered a science; with some of the causes which have prevented the full acknowledgment upon the part of the world of the great obligations under which they are, to the true art of healing, some of which "lie at our own door," and may become the subject of profitable discussion. With the full belief that the world will sooner or later acknowledge the rich boon they have received through medical science, when it stands forth in all its full and glorious proportions; divested of the clouds of error and mystery which have been thrown around it by the ignorant and designing, and as involving questions intimately connected with our future interests and progress, I will address myself to a consideration of our true position.

It is difficult to find a period at which quackery in medicine may be said to have had a commencement. Human nature, since the fall, has been corrupt; and man has continually, from that period, been disposed to seek out many devices by which to avoid the fulfilment of the Divine decree, that, "By the sweat of the brow man should live." And in no age of the world have there been wanting individuals, too scrupulous, to take advantage of opportunities for realizing a subsistence without being very particular to enquire as to the legitimacy of the means; in none of the departments of active life has this disposition been more decidedly manifested than in our own calling. While from the earliest times, there have been individuals who have pursued the investigation of disease with honest and philanthropic purposes, a large number have always been found looking alone to the accumulation of wealth and attainment of position, by practising upon the ignorance and credulity of the world.

The fondness for *mystery* upon the part of the votaries of medicine, and the willingness upon the part of the world to be deceived, perhaps, commenced with Æsculapius (to whom was ascribed the power of raising the dead, and who was worshipped as the God of Medicine) and has descended in no small degree down to our own times. If, the temples which the ancients were wont to raise to him, and in which votive tablets were hung up, on which were recorded the diseases cured, *as they imagined*, by his assistance, are no longer erected to our *modern Gods*, they are able, at least, to record their own deeds, and to raise temples and palaces for themselves from the rich offerings which flow from a perpetuation of the same credulity and ignorance, and doubtless, in this age of the world, *our divinities* prefer the wealth and luxury heaped upon them to all the honors which the ancients awarded to Æsculapius.

It is an interesting question for medical men of the present day to consider why it is that, with all the improvements of the world, with all the enlightenment which has taken place in reference to every department of life, the people still remain, in relation to the true principles involved in the treatment of disease, about as wise as they were in the commencement of the world? Why is it, that the world is as readily imposed upon here in our midst, in this day of light and knowledge, as they were thousands of years ago? Have no *facts* been accumulating to establish our art upon the basis of *science?* are we still groping in original darkness as to the means which the creator designed for the relief of disseased humanity, or is disease a *notion*, and remedy a *humbug?* and, as a legitimate conclusion, does the infinitessimal part of an atom claim rightfully a higher rank as a medicinal agent, than the most efficient article of your "Materia Medica?" Or, after all, have we learned something and the world are unwilling to believe it? This seems to be strange; they are not slow to admit the improvements which have been taking place in all the other departments of life; no man with the most limited knowledge, will deny that we have learned something of natural philosophy, of chemistry, of astronomy, &c., in which we believe the proportion of knowledge to the actual truth is no greater than in our own science, and yet you will find large numbers who exhibit the most acute intelligence upon all other subjects, perfectly ignorant upon this.

To us, as medical men, and as votaries of what we conceive to be a *noble science*, such an exhibition as I have here hastily presented, is discouraging and humiliating in the extreme, and for proof of the truth of the representation, it is only necessary to refer you to your daily experience and to the records of your legislature.

Here in the enlightened State of Georgia, in the midst of the nineteeth century, no discrimination is made in favor of the regular physician who has combined the most thorough opportunities of acquiring an accurate knowledge of his profession, with a proper appreciation of the high obligations resting upon the individual who undertakes to be the *instrument of divinity* in the arrest of disease; and I would here take occasion to acknowledge and remind you, that "we should ever recollect that in our most successful efforts, we are merely the honored instruments of a higher power, for it is God who healeth our diseases and redeemeth our lives from destruction." At the same time, however, that we admit this much, we do not admit that the knowledge and capacity to fit us for such a position is given by *inspiration*—a claim, necessarily set up by some, as the qualification which, they profess, could not possibly have been obtained in any other way.

The Creator has doubtless His fixed laws in relation to disease and its remedies, as a part of the *great system* which He has established for the regulation of this world, and while we will never, probably, acquire perfection in our knowledge of this subject, for this reason, if no other, that "to err is human," we *are* permitted to learn all that is essential to our well being.

But an understanding of these laws can only be acquired by devoting ourselves to their investigation, in the light of the *facts* that have been so long accumulating upon this subject; and we now contend, notwithstanding the mass of error and obscurity which has ever mingled with the actual truth and knowledge which has been established, that we have a *science*; that medicine claims rightfully, to day, a place upon the same platform with the other systems of truth which are fixed, and admitted beyond successful controversy.

That differences of opinion in relation to many points connected with our science have existed from time immemorial, is no more an argument against the *truth* that we have learned, than the almost innumerable sects in religion itself, is an argument against the truth to be found in the inspired volume. Even in reference to that subject, the truth of which (we have the highest authority for saying) is so plain, "that the way-faring man, though a fool, cannot err therein," we have had constantly arising new views and new lights, pointing out some other way to appease an offended God, and to heal the diseases of the soul, than the one fixed by the Creator. It is not, therefore, so surprising that the truth has not been universally admitted in reference to our science, as it might seem to be, without a comparison, with the world's history in regard to some other matters than those which have been referred to. For, while they have been eager to learn whatever facts might have been brought to light, bearing directly and palpably upon the means for acquiring wealth and power in the world from a want of *attention* to medicine, except when they became the subjects of disease, an utter want of capacity to decide upon its merits has been the result, and even where there has been a disposition to investigate, such has been the mystery which has ever *studiously* surrounded our art, that it has been impossible for the superficial observer to discover the truth; and here, it must be confessed, that much of the want of appreciation of our science upon the part of the world, has arisen from the disposition to concealment, and the pretension to oracular wisdom, so much of which has at all times been found among those who have professed to relieve disease.

Indeed, when we consider the amount of impenetrable self-sufficiency, pretension and actual ignorance, which has so often been found in the ranks of the medical profession, we should not be so much surprised at the world's judgment, that stupidity and confidence are the prominent requisites for the position of *Doctor*.

Such has been the facility with which individuals have been introduced into the medical profession, in our own country at least, that a large proportion have ever been unfitted to occupy a place within it; and with the popular idea endorsed in many instances, by our Medical Schools, "that any fool can make a doctor," it is not so surprising that the Farrier and the nurse should be so readily transformed into medical oracles, and have awarded to them the confidence and patronage which is refused to the enlightened physician.

The American system (as it has been called) of manufacturing medical men is now admitted on all hands to be sadly defective, and the primary error, that which lies at the very root of the evil, is the want of preliminary education upon the part of large numbers of those who seek a place in the profession. Prepare the mind by education to appreciate the magnificent field which the science of medicine and its kindred branches unfolds, and it is at once perceived that it is only by the greatest labor and assiduity that its rich fruits can be gathered; and he who has acquired a taste for learning, will thus be stimulated to make the exertions necessary to master truths, a knowledge of which can only be acquired by an earnest, protracted and well directed struggle, while those who are insensible to the pleasures attending the enlightened pursuit of science will retire in disgust from the enterprise.

So long as physicians are made from unprepared material and with such injudicious haste as we too often witness; so long as you find individuals introduced into the profession, who do not themselves appreciate the responsibilities of their high vocation, so long will you find them perverting a noble calling, intended for high and holy uses, to base and dishonorable ends—so long will you find quacks and charlatans claiming the title of physician, and sharing with yourselves the honor and patronage of an undiscerning public. So long as the present system continues, with the inability upon the part of the world to discriminate between the true physician and the unscrupulous pretender, so long will you hear of "vaunted nostrums, of mysterious cures, of infallible systems, the popular credulity with regard to which, in this age, with its wonders of steam and telegraph, and its progress in science, constitutes in our opinion the greatest wonder of them all," unless, indeed, you can enlighten the public mind; but how to do this is a difficult question.

It has been suggested by an intelligent observer, that it is through the channel of *Physiology*, that we are to expect the most. "Could every educated individual be made acquainted with the laws which govern the system, and be prepared to appreciate the remark of Galen, that the study of this science was a perpetual hymn to the honor of Deity, then, indeed, would our art attain a position of influence which has hardly been dreamed of by its most ardent votaries, and rise to such a degree of appreciation by the community, as to sway an influence in the social system, scarcely to be conceived of in the present state of things."

Owing to a want of acquaintance with Physiology, the great science of life, the world expects too much of Physicians; and not finding a cure for every disease with the true man of science, they are driven into the embrace of all sorts of vagaries and follies, set up by the unscrupulous charlatan, or honest ignoramus—whether it be the magic power, formerly attributed to Perkin's points and Berkley's tar-water, or in later times, the absurdities of Hahneman, as exhibited in Homeopathy, or the one idea of Hydropathists, or their opponents, the Heat Doctors, with the almost interminable list of honored patent medicine men, who were formerly only to be found in our large cities, where crowds of thousands could be gathered to see a man go into a quart bottle, but who are now found all over the land, the pigmy representatives of the illustrious Townsend, Jayne, Swaim, &c.

But, while we must be content to concede the right which every man claims, to select his own mode of being doctored, and, while we will not attempt to force the world into the arms of scientific Physicians, and as we cannot exterminate this horde of pseudo-medical men, nor control the action of our law-givers, we may, at least, enjoy what satisfaction we can from the occasional thrusts which they get from the more enlightened and independent portion of the press, as not the least amusing of these just satires upon the absurdity of the pretensions, set up in the flaming advertisements of these patent discoveries, and as an illustration of the inconsistency and utter want of discrimination manifested by English legislators, a full counterpart, to which we have no difficulty in finding upon this side of the water, I would here introduce an amusing article,* with which I have recently met, entitled "Punch on Quackery." It is said that much amusement has been caused by a witty and sensible article in "Punch," on the all-absorbing question of Quacks and Quackery. Punch says that, "A handbill has been forwarded to him, containing a rather choice specimen of the lies with which the quack medicine-mongers endeavor to improve the sale of their pernicious trash. It sets forth that an Indian captain's little girl, aged eight, was in such a state of disease from dropsy, that she was given over by the doctors; her face, a mass of complete ulceration; her teeth were so clenched that her affectionate father had to wrench her mouth open with a wedge in order to force down a dozen pills, No. 2. The very next day she got up, dressed herself, and was discovered sitting on a hard form before a plate of ham and beef." The "rubbish," says Punch, "which the bill in question is intended to puff, is sold, more shame to Mr. Gladstone, under a government stamp. Government is not ashamed *for a consideration* to lend its influence to the quack; some evening, however, the government will be compelled to break up its partnership. In the very handbill to which we allude, and folded round the medicine, the Indian captain is made to say, 'I attribute all my ailings, weakness and diseases to having been bled once, and vaccinated.'

"Under the stamp of the same government," says Punch, "which introduced *the compulsory vaccination act*, most properly

---

*Virginia Medical and Surgical Journal.

enforced under Lord Palmerston's direction, is circulating a notice, that all a person's ailings, weakness and diseases may be attributed to vaccination. Are the people to consider, then, the government lancet *their bane?* Gladstone's pill box *their antidote?* We do think, at least, one thing, that they have a right to complain of what Mrs. Partington would call the vaccinating policy of the government."

As one of the instances of enlightened legislation upon the subject of medicine in our own country, I will refer to an address before the Medical Society of Virginia, by one of the late Presidents of the American Medical Association, in which, he says, "qualification for the practice of medicine is tested in Virginia by the ability to pay ten dollars. If the candidate cannot pass the examination of the sheriff on this branch of science, he is rejected; but if he can, the legal diploma and license are issued, and he is recognized as a doctor of medicine, entitled to all its privileges and immunities, and in the estimation of the law, as well qualified for the practice of his profession, as if he were a graduate of Munich, or a licentiate of Prussia. A candidate for the practice of law, must be certified to be of good moral character, and his learning ascertained and vouched for by three official judges; a minister must pass the examination prescribed by his order; a mechanic must serve an apprenticeship, and at least know the names and use of his tools; but the competency of a doctor of medicine is triumphantly evinced by the payment of ten dollars; a test of ability and learning, which reflects infinite credit on the ingenuity and wisdom of the legislature. It is true, however, that the law, with commendable regard for the public weal, wisely requires him to keep pace with the progress of science, and restrains all delinquency by imposing an annual examination by the *Sheriff*, and the issue of a new diploma from that worthy functionary."

It would not be difficult to find instances of similar legislation all over the land; but however humiliating and discouraging the view here presented to medical men, who are honestly devoting themselves to the proper understanding, and honorable pursuit of a noble profession, we should not despair; but recognizing the principle that "truth will ultimately prevail," diligently labor to master the great truths embodied in our science, and direct our most strenuous efforts to remedy the acknowledged defects of practical medicine. Let not individual members of the profession fold their arms at the apparent hopelessness of the task, but remember that it is only by congregated and organized individual efforts that any reformation can be effected.

Each member of the profession, in his sphere, has his work to perform—not only, in the upright and elevated discharge of his duty in the daily walks of his practice; in the exhibition of principle, above selfishness, looking at all times to the honor of his profession as above the consideration of personal advancement—but, recognizing the great importance of such associations as this which I am now addressing—in which we should gather ourselves, and willingly contribute our aid, in freeing the science and calling from the obstacles which oppose its progress, and sever from our connection and countenance those who, by their course, degrade a noble art into a mere system of humbug and chicanery—labor to enlighten the public mind as to the true nature of the system, promising only what is within the limits of human power to perform, leaving the assumption of divine power to those who profess to accomplish impossibilities—ever extending the hand of welcome to open, fair, and legitimate competition, and independently and fearlessly visiting the frown of non-recognition, as professional brethren, upon the representatives of quackery, in whatever form they may appear—ever being willing to exalt, rather than pull down a professional brother—discarding that miserable jealousy which can only see its own success, in the failure of another—trusting in the great law of supply and demand, to regulate the competition in an overcrowded pursuit.

Let the Atlanta Medical Society, as the representative of true medicine, at this point, feel the responsibilities resting upon them. But, to make it efficient for good, it is desirable and important that the legitimate medical men of the place should present an undivided front; we should number in our ranks all those who are properly recognized as members of the fraternity—we should make the high code of medical ethics, established by the American Medical Association, our creed. None should be willing to be mere drones in the ranks, but each should come up to the discharge of his duty with that zeal and devotion to his profession, which is indispensably necessary, either to his personal advancement, or to the success of any movement which is made for the improvement or honor of his calling.

Truly, there is no science which has done so much for men, and none so little rewarded. No individual can pursue it with any degree of satisfaction, without schooling himself into the practice of a large self-denial; and who does not take, in some degree, a benevolent view of his profession.

Called upon, as we are, to render our services, day and night, through wind and storm, and in a large proportion of cases, without fee or reward; our earnings *ever* proverbially standing in the lowest scale; in many instances, compelled to draw upon funds, for the purchase of medicinal agents, which should be applied to the support of a dependent family; and, that too, without the poor pittance of gratitude in return.

But, gentlemen, I would rather *point you*, upon the other hand, to our work, as a "labor of love," yielding rich fruits, in the consciousness of having been useful in our day and generation—in having discharged our duty in ministering to the wants of suffering humanity, and to the high ends for which we have been placed in the world, than to the injustice and privations visited upon an impoverished profession.

And it is indeed now, more than ever necessary, that we should bestir ourselves; for, notwithstanding the admission of the facts adverted to, which have had a tendency to retard our success, and the proper admission of the great claims which the medical profession has ever had upon the respect and gratitude of the world, our progress is onward. There is, at this day, an amount of talent, energy, and learning, devoted to medical science, which challenges competition with the followers of any other science, or pursuit in life.

Notwithstanding the number of parasites and scullions who are hanging on the outskirts of our army, and have ever been a clog upon our march and a blot upon our escutcheon, there is a noble array of determined and true men, who constitute an invincible phalanx pressing on under the banner of truth and philanthropy, to whom will be eventually erected a monument as enduring as time.

What better calculated to excite the zeal and ardor of a votary of medicine, than a retrospect of the long line of illustrious men who have adorned our profession, and secured for themselves imperishable fame? Who of us can, without honorable pride, and a desire to emulate their great deeds, refer to HARVEY, the immortal discoverer of the circulation of the blood; to JENNER, who has done so much to relieve the world of that horrible pestilence, Small-Pox—an almost entire immunity from which they are only prevented from enjoying by the most inexcusable folly? But time would fail me, to tell of Abernethy, Sydenham, Hunter, Cooper, Bell, Cullen, Louis, and the host of European worthies who have surrounded their names with a halo of glory; and then, in our own land, turn to a review of the labors of the many eminent men, who have been "bright and shining lights" in the galaxy of American Medicine. And, as being equally entitled to commemoration, and as much to the honor of our calling as the most brilliant discovery which has adorned the annals of medicine, I would refer you, with mingled pride and sorrow, to a late and melancholy page in the history of your own State, and that of the desolated city of Savannah, which records a heroism and self-sacrifice upon the part of our profession, rivaling the daring deeds of the most renowned warrior.

High among those who are entitled to the most exalted niche in the temple of medical fame, and to the eternal gratitude of a stricken community, stand Wildman, Wells, Harris, Hartridge, Ellis, Schley, Brantley, Cullen, Saussay, and Gordon—those devoted men, who, in a dark and dismal hour that "tried men's souls," nobly fell in the discharge of their duty, and whose memory should be held aloft, as long as the tide of time shall roll, as a "monument of how great human nature can be."

